# Longitudinal *in vivo* imaging of perineuronal nets

**DOI:** 10.1117/1.NPh.10.1.015008

**Published:** 2023-03-24

**Authors:** Amit Benbenishty, Shany Peled-Hajaj, Venkat Raghavan Krishnaswamy, Hagai Har-Gil, Sapir Havusha-Laufer, Antonella Ruggiero, Inna Slutsky, Pablo Blinder, Irit Sagi

**Affiliations:** a The Weizmann Institute of Science, Department of Immunology and Regenerative Biology, Rehovot, Israel; b Tel Aviv University, Neurobiology, Biochemistry, and Biophysics School, Tel Aviv, Israel; c Tel Aviv University, Department of Physiology and Pharmacology, Sackler Faculty of Medicine, Tel Aviv, Israel; d Tel Aviv University, Sagol School of Neuroscience, Tel Aviv, Israel

**Keywords:** perineuronal nets, extracellular matrix, two-photon imaging, intravital, parvalbumin

## Abstract

**Significance:**

Perineuronal nets (PNNs) are extracellular matrix structures implicated in learning, memory, information processing, synaptic plasticity, and neuroprotection. However, our understanding of mechanisms governing the evidently important contribution of PNNs to central nervous system function is lacking. A primary cause for this gap of knowledge is the absence of direct experimental tools to study their role *in vivo*.

**Aim:**

We introduce a robust approach for quantitative longitudinal imaging of PNNs in brains of awake mice at subcellular resolution.

**Approach:**

We label PNNs *in vivo* with commercially available compounds and monitor their dynamics with two-photon imaging.

**Results:**

Using our approach, we show that it is possible to longitudinally follow the same PNNs *in vivo* while monitoring degradation and reconstitution of PNNs. We demonstrate the compatibility of our method to simultaneously monitor neuronal calcium dynamics *in vivo* and compare the activity of neurons with and without PNNs.

**Conclusion:**

Our approach is tailored for studying the intricate role of PNNs *in vivo*, while paving the road for elucidating their role in different neuropathological conditions.

## 1 Introduction

Recent years have witnessed a rapidly growing number of studies focusing on the intriguing functions of perineuronal nets (PNNs), unique specialized polysaccharide-based extracellular matrix structures.^1^^–^^4^ PNNs form during the end of the critical period and are important mediators of neuronal plasticity, learning, and memory.^5^ Furthermore, it has been suggested that PNNs also protect neurons from oxidative stress^6^ and damage elicited by activated microglia,^7^ and mediate transmission of pain signals from the spinal cord.^8^ These extracellular nets enwrap mostly inhibitory parvalbumin-positive (${\mathrm{PV}}^{+}$) interneurons and are proposed to regulate their activity.^9^ Accordingly, they affect the synchronization of neuronal oscillations,^10^^–^^12^ thus modulating consequent information processing and consolidation. The late Nobel laureate Roger Tsien postulated that PNNs are the primary structures for holding life-long memories,^13^ pointing to their stability as a key pillar in the basis of this hypothesis. Related to this, it has been shown that Alzheimer’s patients lose about two-third of their PNNs.^14^ The view of PNNs as stable structures has been challenged by recent studies that found rapid changes in the number of PNNs following fear conditioning and extinction of learning.^15^ However, to better understand when and how PNNs form and degrade, the ability to capture these dynamic processes is essential.

Currently, the role of PNNs is studied using *ex vivo* stainings to quantify their distribution^16^ and structure^17^ or in combination with electrophisiological recordings in acute slices^9^ to understand their role in activity. Unfortunetly, *ex vivo* recordings from tissue slices can be performed only within a short-time frame after their preparation and are only performed on partial neuronal systems with incomplete microenvironments.^18^ Enzymatic degradation of components of the PNNs, such as the chondroitin sulfate proteoglycans (CSPGs)^11^ or hyaluronic acid,^19^ is another prevalent approach for studying their role. However, due to the inability to follow the dynamic process of degradation and assembly of PNNs *in vivo*, the scope of this effective tool remains limited in its use for correlative assesment of phenotypic outcomes.^13^

Hence, although existing experimental approaches have advanced our understanding regarding the important roles of PNNs in brain function, their assesment of longitudinal and dynamic processes, such as sensory processing, learning, memory, and neuroprotection, is limited.^4^^,^^20^ We set here to address the unmet need for advanced methods allowing to quantify dynamic processes associated with PNNs and PNN-positive (${\mathrm{PNN}}^{+}$) neurons within their native microenvironments. Nearly three decades ago, a study showed that stereotactic intracranial injections of biotinylated Wisteria floribunda agglutinin (WFA) in rats, followed by *ex vivo* streptavidin incubation, resulted in specific staining of PNNs.^21^ We build upon this pioneering work, which was never used *in vivo*, and introduce a straightforward approach for direct labeling of PNNs in mice via intracranial injections of fluorescently labeled WFA. This procedure is particularly well-suited for *in vivo* imaging, as demonstrated here with two-photon imaging. This approach allows, for the first time, to longitudinally image PNNs, observe the dynamics of PNN breakdown and *de novo* synthesis, as well as to segregate and monitor neuronal activty of ${\mathrm{PNN}}^{+}$ cells, all with high specificity and with subcelluar resolution in a minimally invasive manner.

**Fig. 1:**
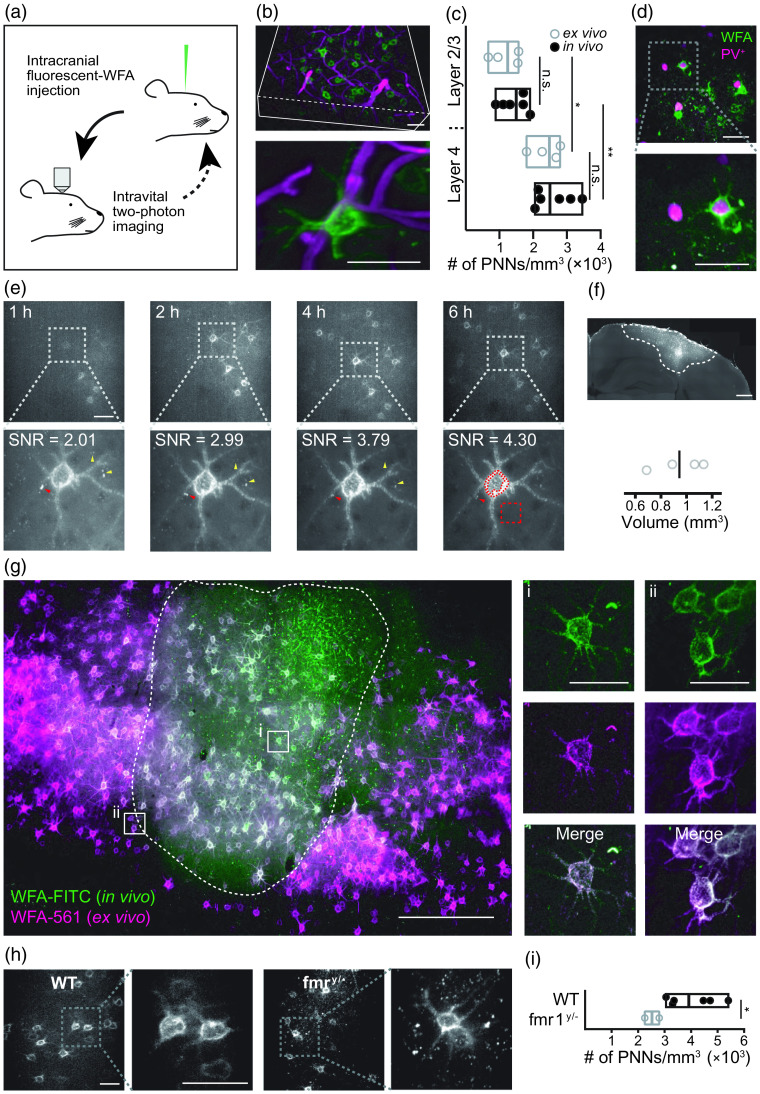
Intracranial injection of fluorescent-WFA rapidly and robustly labels PNNs for longitudinal intravital imaging. (a) A diagram representing the workflow used in the study. Stereotactic injection of fluorescent-WFA was followed by intravital two-photon imaging for measuring activity in awake animals, for observing PNNs, or for monitoring enzymatic degradation and synthesis of PNNs. Our system allowed multiple injections and longitudinal imaging. (b) Injection of FITC-conjugated WFA allowed *in vivo* imaging of PNNs in high resolution with minimal unspecific staining. Top image represents a 3D-reconstruction of a partial stack (200 to 350  μm deep into the cortex), and bottom image represent a z-projection of a single PNN covering the neuron soma and initial segments of the dendrites. Green, WFA and magenta, blood vessels. (c) The number of PNNs counted in layers 2 to 4 *in vivo* (n=4 mice) was similar to the number of PNNs counted in histological sections (n=3 mice; layers 2/3, p=0.8883; layer 4, p=0.9094), whereas in both *in vivo* and *ex vivo*, we found more PNNs in layer 4 compared with layers 2/3 (*in vivo*, p=0.0022; *ex vivo*, p=0.0104; ordinary one-way ANOVA, $F_{\left(3,16\right)}=10.82$, p=0.0004). Box plots represent median with min–max values. (d) PNN staining (green) in ${\mathrm{PV}}^{+}$ tdTomato (magenta) mice. (e) PNNs were visiable *in vivo* as early as 1 h following intracranial injection of WFA-FITC, and SNR more than doubled in the first 6 h and remained stable thereafter. SNR was calculated based on maximum intensity z-projections as median value of free-form encompasing the PNN over median value of square in the background, as indicated in the bottom right image. Red arrowheads point to specific staining that improved over time and yellow arrow heads point to nonspecific staining that reduced or disappeared over time. (f) Quantification of the volume stained by a single injection of 1.5  μl WFA-FITC at 50 to 500  μm from pial surface, 4 days following injection. (g) Histological analysis of brains 60 days following WFA-FITC (green) injection revealed that all PNNs around the injection site were labeled, as indicated by *ex vivo* staining (magenta) with WFA-Alexa Fluor 561. (h) *In vivo* PNN imaging allowed visualizing PNNs in Fmr1-knockout mice. (i) Quantification of the PNNs indicated there was significantly less PNNs in Fmr1-knockout mice compared with WT mice (Welch’s two-sided unpaired t-test, t(4)=3.159, p=0.0277). Box plots represent median with min–max values. Images represent a 30-μm maximum intensity z projection. Scale bars are 50  μm for (b)–(e), and (h), 200  μm for (f)–(g), and 20  μm for insets in (g). See also Figs. S1–S3 in the Supplementary Material and Videos 1–4 (Video 1, MOV, 11.4 MB [URL: https://doi.org/10.1117/1.NPh.10.1.015008.s1]; Video 2, MP4, 1.44 MB [URL: https://doi.org/10.1117/1.NPh.10.1.015008.s2]; Video 3, MOV, 10.9 MB [URL: https://doi.org/10.1117/1.NPh.10.1.015008.s3]; and Video 4, MOV, 11.2 MB [URL: https://doi.org/10.1117/1.NPh.10.1.015008.s4].).

**Fig. 2:**
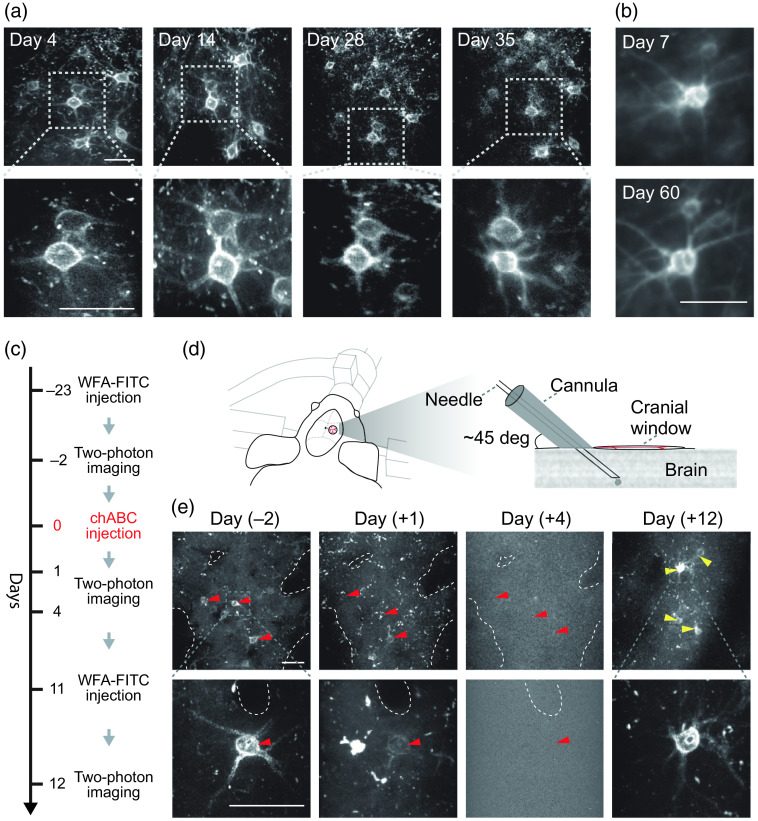
*In vivo* labeling of PNNs proves to be stable, while enabling monitoring degradation and *de novo* synthesis of PNNs. (a) Longitudinal imaging of the same PNNs labeled with WFA-FITC over a period of 35 days. No loss of signal was detected. (b) *In vivo* injection of WFA-Alexa594 stained PNNs specifically and was stable for at least 60 days following injection. (c) Timeline for *in vivo* monitoring of degradation and *de novo* synthesis of PNNs. (d) A sketch representing the window and cannula preparation for multiple intracranial injections. (e) Longitudinal imaging with multiple injections of WFA-FITC and chABC allowed monitoring the enzymatic destruction process of the PNNs and their reconstruction. “Day (X)” indicates the time relative to chABC injection. Red arrowheads point to PNNs that underwent degradation and yellow arrowheads point to newly formed PNNs. Images represent a 30-μm maximum intensity z projection. Scale bars are 50  μm.

WFA is a lectin that binds to the glycosaminoglycan chains of the CSPGs found abundantly in the PNNs. Staining with WFA is among the most used approaches for visualizing PNNs *in vitro*,^22^^,^^23^ in histological sections,^16^ and in acute slices.^9^ Expanding this experimental strategy for *in vivo* applications, we injected either fluorescein isothiocyanate (FITC)-, Atto 590-, or Alexa Fluor™ 594 (Alexa594)-conjugated WFA into the cortical parenchyma of mice implanted with a cranial window, followed by intravital two-photon imaging [Fig. 1(a)]. We used a monomeric version of WFA to minimize its interference with physiological processes and show that the *in vivo* labeling of PNNs is robust (Fig. 1 and Figs. S1–S3 in the Supplementary Material) does not affect intrinsic electrophysiological properties of stained neurons (Fig. S4 in the Supplementary Material) or enzymatic degradation (Fig. S5 in the Supplementary Material), rapid [Fig. 1(e)], specific [Fig. 1(g) and Figs. S1–S3 in the Supplementary Material], and stable [Figs. 2(a) and 2(b)], making it ideal for longitudinal imaging. We start by demonstrating the use of this tool to characterize PNNs in a neurodevelopmental disease mouse model of fragile X syndrome [Figs. 1(h) and 1(i)]. We chose fragile X syndrome as a model disease, as the number of PNNs have been repeatedly shown to decrease in multiple studies and were shown to have an important role in this disease (see Ref. 4 for a review).

Next, we monitor their stability over time [Figs. 2(a) and 2(b)] as well as their degradation and reconstitution^19^ following chondroitinase ABC (chABC) injection [Figs. 2(c)–2(e)]; this also strengthens the notion that the staining agent does not interfere with enzymatic degradation *in vivo*.

**Fig. 3:**
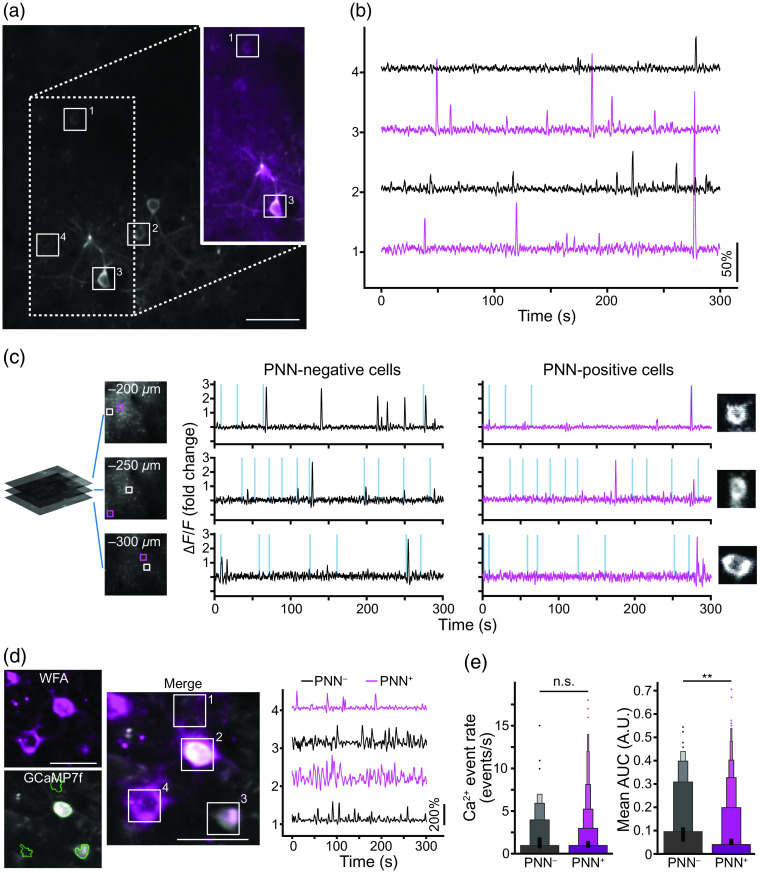
Intravital calcium imaging of mice with labeled PNNs. (a) A representative GCaMP6s-labeled field of view (FOV) with markings of some of the detected active components. The 290×290  μm FOV was captured at 30 Hz, with the depicted image being 1000 frames average for display purposes only. Inset – 30 frames averaged image of ${\mathrm{PNN}}^{+}$ cells (WFA-Atto590; magenta) overlaying the GCaMP image (grayscale). (b) Representative ΔF/F traces of the marked cells with a PNN (magenta) and without a PNN (black). (c) Representative ΔF/F traces of ${\mathrm{PNN}}^{-}$ (left; black) and ${\mathrm{PNN}}^{+}$ (right; magenta) neurons at different cortical depths (measured from pia) in a mouse injected with WFA-FITC and RCaMP7. Vertical lines in the traces indicate the timing of whisker stimulation and are scaled to 300% ΔF/F change. (d) A representative FOV of ${\mathrm{PV}}^{+}$ cells expressing labeled with WFA-Alexa594 (magenta) and GCaMP7f (gray) with marking of some of the detected active components (as detected by CaImAn), with their corresponding ΔF/F traces of the marked cells with a PNN (magenta) and without a PNN (black) (see Video 5, MOV, 1.52 MB [URL: https://doi.org/10.1117/1.NPh.10.1.015008.s5]). (e) The calcium transient event rate in the barrel cortex was similar between ${\mathrm{PNN}}^{+}$ and ${\mathrm{PNN}}^{-}$ PV cells, while the mean AUCs of the ΔF/F traces were significantly higher in ${\mathrm{PNN}}^{+}$ PV cells compared to ${\mathrm{PNN}}^{-}$ cells in the same mouse (n=3 mouse, 147 ${\mathrm{PNN}}^{+}$ cells and 1025 ${\mathrm{PNN}}^{-}$ cells, unpaired two-tailed student t-test, t=2.6066, p=0.0092). Moreover, the variance of the mean AUCs of ${\mathrm{PNN}}^{-}$ cells was significantly larger compared to ${\mathrm{PNN}}^{+}$ PV cells (Levene’s test, p=0.0092). Data were averaged to 1 fps for display purposes only. Scale bars are 50  μm.

We further demonstrate the feasibility of simultaneously combining calcium imaging with this labeling approach to monitor the activity of PNN-enwrapped neurons [Figs. 3(a)–3(c)], paving the road for a direct study *in vivo* of ${\mathrm{PNN}}^{+}$ and PNN-negative (${\mathrm{PNN}}^{-}$) PV interneurons [Figs. 3(d) and 3(e)].

## 2 Results

Injection of a commercially available WFA-FITC lectin to the somatosensory cortex resulted in specific PNN staining [Fig. 1(b) and Video 1]. Following fluorescent-WFA injection, PNNs were visible *in vivo* in layers 2/3 (75 to 200  μm below the pial surface) and more abundantly in layer 4 (200 to 400  μm; n=6 mice; 1440±398.80 versus 2630±577.10 WFA-positive cells, mean ± SD, p=0.0022; Figs. S1 and S2 in the Supplementary Material). These values were comparable to the number of PNN-bearing cells observed in the same location following *ex vivo* staining [n=4 mice; 1185±445.90 in layers 2/3 versus 2391±452.60 in layer 4, mean ± SD, p=0.0104; Fig. 1(c)], matching previously reported values measured *ex vivo*.^16^^,^^24^ Similarly, ${\mathrm{PNN}}^{+}$ cells were only sporadically found in layer 1, while some large blood vessels were partially stained (Fig. S2 in the Supplementary Material and Video 1).

Past *ex vivo* histological studies pointed to the fact that most of the PNNs enwrap ${\mathrm{PV}}^{+}$ inhibitory neurons while not all ${\mathrm{PV}}^{+}$ cells are enwrapped by a PNN.^25^ To demonstrate the feasibility of using *in vivo* staining of PNNs to segregate between these ${\mathrm{PV}}^{+}$ populations, we injected WFA-FITC into brains of transgenic mice that express tdTomato in most of these cells (i.e., ${\mathrm{PV}}^{+}$-tdTomato). This approach allowed to clearly differentiate between ${\mathrm{PV}}^{+}$ cells with and without PNNs [Figs. 1(d), 3(d), and 3(e) and Videos 2 and 3].

Remarkably, PNNs were visible within 1 h following injection of fluorescent-WFA, and after 2 h we did not detect staining of new components. Unbound fluorescent WFA lectin was washed away rapidly or diluted in the extracellular fluid, whereas the bound lectin remained intact. Consequently, the signal-to-noise ratio (SNR) of the staining improved by twofolds reaching a maximum within 6 h following the injection of fluorescent WFA [Fig. 1(e)].

Injection of 1.5  μl of WFA-FITC in a single location at depths ranging between 50 and 500  μm from pial surface resulted in staining of PNNs in an average volume of $943.10\;{\mu m}^{3}$ (n=4; $\mathrm{SD}=198.84\;{\mu m}^{3}$). The fluorescent WFA diffused in the lateral axis up to ∼2  mm and reached as deep as ∼1.75  mm [Fig. 1(f)].

Histological sections of brain tissues were analyzed 60 days following *in vivo* injection of WFA-FITC [Fig. 1(g) and Video 4]. This analysis confirmed that PNNs were robustly, specifically, and stably labeled. *Ex vivo* staining of the sections with WFA conjugated to Alexa 561 [Fig. 1(g)] or with antiaggrecan antibody (Fig. S3 in the Supplementary Material) proved that the *in vivo* staining was specific to PNNs and stained all the PNNs in the injection area. To test the use of our approach in a disease model, we monitored PNNs *in vivo* in a mouse model of fragile X syndrome [i.e., Fmr1-knockout mice; Figs. 1(h) and 1(i)] and found considerably less PNNs in these animals (p=0.0277), similar to previously described results from *ex vivo* experiments^4^ highlighting the use of our method to study *in vivo* this and other pathologies related to PNNs.

Importantly, labeling of the PNNs with either WFA-FITC, WFA-Atto590, or WFA-Alexa594 was stable, enabling longitudinal imaging of the same PNNs at subcellular resolution over more than 8 weeks [Figs. 2(a) and 2(b)]. Together, these findings indicate that the presented approach allows reliable monitoring PNNs very early after injection of fluorescent WFA as well as for weeks to follow.

*In vivo* detection of PNNs is critical for monitoring dynamic processes, such as degradation, synthesis, and activity. To this end, we monitored enzymatic degradation and reconstitution of PNNs *in vivo* [Figs. 2(c)–2(e)], a phenomenon that to date was studied only *ex vivo*.^19^ Although *in vivo* enzymatic degradation with chABC occurred within a similar time frame as previously reported,^11^^,^^26^^,^^27^ it is still possible that the labeling interferes with intrinsic enzymatic processing, carried out by proteases such as matrix metalloproteinases (MMPs) and a disintegrin and metalloproteinase with thrombospondin motifs (ADAMTSs).^4^^,^^28^^–^^30^ To test this, we assessed whether ADAMTS4, a proteoglycanase found in the brain in physiological and pathological conditions,^31^ can degrade labeled PNNs. We found that PNNs labeled with WFA-FITC were degraded by low concentration (10 nM) of the enzyme, indicating that the labeling itself does not significantly affect the stability of PNNs (Fig. S5 in the Supplementary Material).

Longitudinal monitoring of neuronal activity in behaving mice is another essential aspect for understanding the role of PNNs. To demonstrate the applicability of our method in this context, we conjugated a monomeric WFA lectin to Atto 590 (see Sec. 4) and injected it together with an AAV-hSyn-GCaMP6f virus, or injected WFA-FITC together with an AAV-hSyn-RCaMP7.01, to induce expression of the calcium indicator in all neuronal populations in the injected area. We show the activity of single neurons and correlate it with the presence of PNNs [Figs. 3(a)–3(c)]. Next, we investigated the difference in activity dynamics of ${\mathrm{PV}}^{+}/{\mathrm{PNN}}^{+}$ compared with ${\mathrm{PV}}^{+}/{\mathrm{PNN}}^{-}$ interneurons by injecting WFA conjugated to Alexa Fluor™ 594 (see Sec. 4) together with AAV-floxed-GCaMP7f into ${\mathrm{PV}}^{\mathrm{cre}}$ mice [Fig. 3(d)]. First, to ensure that the labeling of the PNNs does not alter the activity of the enwrapped neurons, we quantified the electrophysiological properties of labeled and nonlabeled PV-interneurons in primary cultures (Fig. S4 in the Supplementary Material). Electrophysiological measurements indicated that WFA labeling did not affect firing rates (p=0.6456), action potential threshold (p=0.5305), action potential half width (p=0.4920), or membrane resistance (p=0.8377). Notably, other studies have used WFA for labeling PNNs during electrophysiological recordings in acute slices and cultures and did not report any methodological artifacts;^9^^,^^32^ further justifying the approach presented here. *In vivo*, we found that while the detected calcium transient event rate in the barrel cortex was similar in ${\mathrm{PV}}^{+}/{\mathrm{PNN}}^{+}$ and ${\mathrm{PV}}^{+}/{\mathrm{PNN}}^{-}$ interneurons, the mean and median area under the curve (AUC) of ${\mathrm{PV}}^{+}/{\mathrm{PNN}}^{+}$ were larger [p<0.01 and p<0.05, respectively; Fig. 3(e)]. Furthermore, the variance between the neurons’ means and medians AUC was much larger in cells without PNNs (p<0.01 and p<0.05, respectively). The results herein are not directly comparable with previous studies that investigated the regulating role of PNNs on PV activity, because they used electrophysiological recordings to measure the effects of removal of PNNs or one of their components.^33^ Complete removal of PNNs by enzymatic degradation or removal of specific PNN components by genetic manipulations resulted in most cases in the reduction of firing rate and increased spiking variability. Although these are useful tools, they neither allow to elucidate if PNNs enwrap neurons with intrinsically different activity characteristics nor do they allow to elucidate any mediating role of PNNs on such activity under physiological conditions. Future studies should combine *in vivo* labeling of PNNs and activity monitoring (using calcium or voltage imaging or electrophysiological reordering) with manipulations on PNNs to tackle these critical gaps in our current knowledge.

## 3 Discussion

Overall, we demonstrate a simple and robust method for specifically detecting PNNs *in vivo* using commercially available reagents. We prove this staining is specific and stable over time, enabling longitudinal *in vivo* imaging without affecting the underlying physiological properties or the efficacy of enzymatic degradation of the PNNs. We also track longitudinal and dynamic processes, such as degradation and reconstitution of PNNs in normal and pathological conditions. We further demonstrate that this approach is compatible with the most common calcium imaging indicators (i.e., GCaMP and RCaMP) and can be used to monitor activity of ${\mathrm{PNN}}^{+}$ neurons in a comparative manner within their natural environments while maintaining their integrity and physiological interactions. Utilizing this labeling approach, we provide initial evidence that PNNs are found around ${\mathrm{PV}}^{+}$ interneurons with distinct activity characteristics, including apparent increased and more stable firing properties. Future work should investigate whether the PNNs enwrap two, or more, distinct PV populations or that the presence of PNNs themselves changes the activity of the cells through processes, such as stabilization of synapses^2^ or binding of metabolites and growth factors.^4^

The PNN-labeling approach presented herein involves an intracranial injection and therefore has some limitation compared with genetically encoded labeling, such as induction of local and transient inflammation or the need of repeated injection for *de novo* synthesis studies [as done in Figs. 2(c)–2(e)]. However, transgenic models that allow *in vivo* PNN visualization are not yet available. In addition, *in vivo* imaging with high spatial resolution at depths, PNNs are present necessitates implantation of a cranial window, which, by itself, unavoidably induce similar transient inflammation. Furthermore, extrinsic labeling approaches also have advantages over transgenic models, such as reduced costs, time, and animal breeding requirements, as well as providing improved labeling robustness. We foresee that like the historical development of calcium and membrane potential indicators, vast amounts of knowledge can be gained using a synthetic indicator (i.e., WFA labeling) until their genetically encoded counterparts become available and mature while we attain the performance level of the synthetic ones, a process that can take several years. With the growing interest in PNNs, we expect that this approach will become a groundbreaking tool for studying the intricate physiological role of PNNs *in vivo* in health and disease. We expect the application of the technique presented here to elucidate the role of PNNs in regulating activity of PV cells, the interaction of PNN with glial cells and more, during development and disease progression.

## 4 Materials and Methods

### 4.1 Experimental Design

To test the feasibility of *in vivo* labeling and imaging of PNNs, we injected fluorescently labeled WFA; and at different time points, we imaged WT, PV-tdTomato, and Fmr1-knockout mice implanted with cranial windows. To image activity of ${\mathrm{PNN}}^{+}$ and ${\mathrm{PNN}}^{-}$ cells, we injected fluorescently labeled WFA together with a fluorescent calcium indicator. To assess PNN degradation and *de novo* synthesis, we injected WFA-FITC, followed by chABC and WFA-FITC, as detailed below.

### 4.2 Animals

All studies were approved by the Tel Aviv University ethics committee for animal use and welfare and were in full compliance with Institutional Animal Care and Use Committee guidelines. Male and female mice (P42-120; C57BL6 wild-type or transgenic mouse lines with various genetic backgrounds) were used. Animals were housed under standard vivarium conditions (22°C±1°C, 12-h light/dark cycle, with *ad libitum* food and water). For the euthanasia of animals, we used excess ${\mathrm{CO}}_{2}$ or sodium pentobarbital (200  mg/kg; i.p.).

Transgenic animals used in this study included $B6.\mathrm{Cg}\text{-}\mathrm{Gt}(\mathrm{ROSA}){26\mathrm{Sor}}^{\mathrm{tm}9(\mathrm{CAG}\text{-}\text{tdTomato})\mathrm{Hze}}/J$ crossed with $B6.\mathrm{Cg}\text{-}\mathrm{Gt}(\mathrm{ROSA}){26\mathrm{Sor}}^{\mathrm{tm}9(\mathrm{CAG}\text{-}\text{tdTomato})\mathrm{Hze}}/J$ mice to create PV-tdTomato mice for visualization of ${\mathrm{PV}}^{+}$ cells *in vivo*, B6.129P2-Pvalbtm1(cre)Arbr/J (${\mathrm{PV}}^{\mathrm{cre}}$) mice for studying activity specifically in ${\mathrm{PV}}^{+}\;\text{cells}$ *in vivo*, and $B6.{129P2\text{-}\mathrm{Fmr}1}^{\mathrm{tm}1\mathrm{Cgr}}/J$ as a disease model of fragile X syndrome.

### 4.3 WFA Conjugation

The conjugation of Atto590 and Alexa594 with WFA was achieved through amine-reactive chemistry using the free amine groups in the WFA lectin. Freshly prepared 0.4 mg of Atto590 NHS ester (79636 and 68616, dye and protein labeling kit, respectively, Sigma Aldrich) or a full vial of Alexa Fluor™ 594 NHS ester (A10239, protein labeling kit, Thermo Fisher Scientific) dyes in DMSO was mixed with 3 mg or 1 mg, respectively, at a concentration not <2  mg/ml (L8258, Sigma Aldrich) in bicarbonate buffer (pH 8.3). The reaction mixture was incubated in dark in a rotating mixer for 3 h at room temperature. The solution was then laid over on a silica column preactivated with phosphate buffered saline (PBS). The dye-conjugated WFA and the free dye were eluted with sodium phosphate buffer and were collected separately. Conjugation efficacy was tested by staining brain slices (1:150) and *in vivo* following intracranial injections (Fig. S1 in the Supplementary Material).

### 4.4 Injection of Fluorescently Conjugated WFA

Mice were anesthetized with isoflurane (5% for induction, 1.5% thereafter) and given dexamethasone (intramuscular, 2  mg/kg) and carprofen (intraperitoneally, 5  mg/kg). The animals’ core body temperature was maintained at 37°. A 3-mm diameter craniotomy was made as previously described (window center was at 2 mm posterior and 3.5 mm lateral from bregma).^34^ Before placement of the cover glass, the animals were injected with a conjugated-WFA through the cranial window into the barrel cortex (3 to 3.25 mm right, 1 to 1.5 mm posterior relative to bregma). ${\mathrm{PV}}^{+}$-tdTomato mice were injected with FITC-conjugated WFA (FL-1351, Vector Laboratories). C57Bl/6J animals were injected with FITC-conjugated WFA together with an AAV-virus expressing RCaMP7.01 [hSyn1-chI-RCaMP1.07-WPRE-SV40p(A), v224-1; titer: ∼6.4×1012; Viral Vector Facility, ETH Zurich] or with Atto590-conjugated WFA together with an AAV-virus expressing GCaMP6s (Syn.GCaMP6s.WPRE.SV40, AV-1-PV2824; titer: ∼3.6×1012; UPenn Vector Core) for monitoring activity. To monitor activity in ${\mathrm{PV}}^{+}$ interneurons with and without PNNs, ${\mathrm{PV}}^{\mathrm{cre}}$ mice were injected with Alexa594-conjugated WFA together with an AAV-virus expressing floxed GCaMP7f [ssAAV-1/2-hSyn1-chI-dlox-jGCaMP7f(rev)-dlox-WPRE-SV40p(A), v319-1; titer: ∼6.4×1012; Viral Vector Facility, ETH Zurich]. To avoid possible interference with the distribution and uptake of the calcium indicators, we injected the virus and waited for 15 min before the lectin was injected adjacently. We allowed expression of the indicators for 3 to 5 weeks following viral injection prior to performing the calcium imaging experiment.

Briefly, fluorescently conjugated WFAs were first diluted (1:3 ratio) with artificial cerebrospinal fluid (WFA-FITC) or PBS (WFA-Atto590/Alexa594-WFA), and aspirated in 4  μl aliquots into glass micropipettes of 1-mm outer diameter and a 0.5-mm inner diameter (BF100-50-10, Sutter Instrument), which were pulled using a micropipette puller (P-2000, Sutter Instrument) to a final tip thickness of 20 to 45  μm. The WFA and WFA-virus solutions were injected into 1 to 3 injection sites using a different micropipette per each injection site. The solutions were pressure injected using a PicoSpritzer II (Parker, Hannifin Corporation) at a $0.15\;\mu l\;\min^{-1}$ injection rate. In each site, ∼1.5  μl were injected starting at a depth of 500  μm and up to 50  μm below the pial surface, with 10 injections every 50  μm. For repeated injections of WFA, a custom-made miniature cannula was placed in a ∼45-deg angle directly proximal to the cover glass [Fig. 2(d)]. A 35G beveled needle (NF35BV, NanoFil) mounted on a 10-μl NanoFil syringe was inserted into the brain through the cannula and WFA-FITC or chABC was injected. For WFA-FITC, 3  μl of solution were injected at a rate of 0.1  μl/min starting at a depth of 500  μm and up to 50  μm below the pial surface, with an injection of 0.5  μl every 75  μm. For chABC, 1  μl of solution (C3667, Sigma-Aldrich; 50  U/ml; 0.1  μl/min) was injected through the cannula in a single injection at a depth of ∼300  μm.

### 4.5 Intravital Two-Photon Imaging

For intravital imaging recordings, animals were head fixed, either anesthetized when WFA staining was imaged for morphological purposes (Figs. 1 and 2, Fig. S1 in the Supplementary Material, and Video 1) or awake while standing on a treadmill when imaging neuronal calcium dynamics (Fig. 3). For blood vessel imaging [Fig. 1(b)], Texas Red dextran (D3328, Thermo Fisher Scientific) was injected immediately before imaging. Imaging was done using a modified version of an MOM two-photon microscope (Sutter Instrument Company’s Movable Objective Microscope), equipped with an 8-kHz resonant-galvanometric scanning unit. The laser source was an 80-MHz Ti:Sapphire laser (Chameleon Ultra II, Coherent Inc.), tuned to 800 nm when recording Atto590- and FITC-conjugated WFA signals, 940 nm when recording GCaMP6s calcium activity and tdTomato signals, and 780 nm when capturing Alexa594-conjugated WFA signals. For recording RCaMP7 calcium activity, we illuminated the sample with the Orange HP10 fiber laser (Menlo Systems GmbH, Martinsried, Germany) at a wavelength of 1040 nm and a 160-MHz repetition rate. We used either a 10× (0.6 NA, Olympus) or a 25× (0.95 NA, Leica) water-immersion objective. Collected light was directed into the detection system by dichroic mirrors (BrightLine FF735-Di01-25×36, BrightLine FF01-625/90-25 25×25 by Semrock, and 565dcxr by Chroma Technology Corporation) and a bandpass filter (525/70-2P, Chroma Technology Corporation). The detectors consisted of two GaAsP photomultiplier tubes (H10770PA-40SEL, Hamamatsu Photonics K.K.) whose output was preamplified (TA1000B-100-50, Fast ComTec GmbH) and discriminated (MCS6A-2T8 Fast ComTec GmbH) before being digitized by a fast sampler (FlexRIO PXIe-1073 with the 5734-adapter module, 120 MHz sampling, National Instruments). The discrimination process, described by Har-Gil et al.,^35^ reduces the background noise and improves the SNR.

For calcium imaging, preformed in layer 4 of the barrel cortex (i.e., 200 to 400  μm below pial surface), whisker stimulation was induced using a custom-built air-puff system, delivering air puffs at a rate of 5 Hz every 13 to 17 s with a random interstimulus interval, either directed toward the whisker pad or directed away from them (to serve as an auditory control). Time lapse recordings of calcium activity were analyzed using CaImAn,^36^ a computational framework designed to conduct motion correction, source extraction, and denoising on imaging data. Image processing of the displayed images captured *in vivo* was limited to Gaussian kernel smoothing and overlaying using Fiji (version 2)^37^ with the sole purpose of improving display. Reconstructions [Fig. 1(b) and Videos 2, 3, and 4] were done using Imaris (versions 9.5); raw data were used for analysis of activity in ${\mathrm{PNN}}^{+}$ and ${\mathrm{PNN}}^{-}$ PV cells [Figs. 3(d) and 3(e)]. We followed a custom macrodetailed in Fig. S7 in the Supplementary Material to mask the PNN channel to automatically and blindly decipher between these two cell populations.

### 4.6 Tissue Processing and Immunostaining

For histological analyses, mice were transcardially perfused with PBS-heparin followed by 4% paraformaldehyde. Brains were extracted and fixed for 24 h at 4°C, followed by 24 h in 30% sucrose for cryoprotection. Sixty-micrometer sections were cut using a freezing microtome (SM 2000, Leica) and processed as free-floating brain sections. Sections were blocked for 1 h in blocking solution (5% goat serum, 0.2% Triton-X in PBS) at room temperature, followed by overnight incubation at 4°C with primary or conjugated antibodies in blocking solution. The primary antibodies used were biotinylated WFA (1:500, L1516, Sigma-Aldrich) and rabbit antiaggrecan (1:200, ab1031, Abcam). Conjugated antibodies were WFA-FITC (1:100, FL-1351, Vector Laboratories) and WFA-Alexa594 (1:150, see WFA conjugation section). The sections were then washed and incubated for 2 h at room temperature with the corresponding secondary antibodies in blocking solution—streptavidin Alexa Fluor 594 (1:1000, S11227, Invitrogen) and goat antirabbit Alexa Fluor 594 (1:1000, A11037, Invitrogen), followed by DAPI (1:1000; 0215757405, 363 MPbio) for nuclei visualization. Images of the sections were obtained using a Leica SP8 confocal microscope mounted with an 40× water-immersion objective (1.1 NA) [Fig. 1(g), Fig. S3 in the Supplementary Material, and Video 4] or a Leica DMi8 inverted wide-field microscope mounted with an 20× objective (0.4 NA) [Figs. 1(c) and 1(e) and Figs. S1A and S2 in the Supplementary Material].

For quantification, we used at least three sections from each mouse.

### 4.7 Enzymology

C57BL6 wild-type mice were transcardially perfused with PBS-heparin. Brains were extracted, placed in optimal cutting temperature compound (OCT) molds on dry ice, and stored in −80°C. Fifteen-micrometer sections were cut using a cryostat (CM1950, Leoca) and chosen slices that included the somatosensory cortex were placed on poly-L-lysine imaging plates (81148, IBIDI). The plates were stored in −80°C until use. Two plates were taken and washed in PBS for 5 min. Each plate was treated with blocking buffer (20% donkey serum, 0.2% Triton in PBS) and incubated in room temperature for 1 h. Samples were then stained with WFA-FITC (VE-FL-1351-2, Vector Labs, 1:200) and DAPI (D9542, Sigma Aldrich, 1:1000). Samples were incubated overnight at 4°C followed by three washes with PBS. Samples were washed twice in TNC buffer (50  μM of Tris with pH 7.5, 5  μM of ${\mathrm{CaCl}}_{2}$, 100  μM of NaCl, and 0.05% Brij-35 in DDW). Then one sample was treated with 500  μl of TNC, whereas the other was treated with 500  μl of 10 nM of ADAMTS4 (CC1028, Sigma Aldrich). Both samples were imaged at intervals of ∼10  min in a fluorescent spinning disc microscope (Nikon Eclipse Ti2, 20× magnification). The total intensity and covered area of resulting images were quantified using Amira software (version 2022.1, Thermo Fisher Scientific). To account for methodological-related changes in intensity (e.g., bleaching, movement of the plates, laser intensity fluctuations, etc.), each time point was normalized to baseline and then WFA and DAPI intensities of the ADAMTS4 samples were compared with the TNC (control) samples. This experiment was replicated and both experiments were used for statistical analysis.

### 4.8 Primary Hippocampal Cultures and Patch Clamp Electrophysiology

Primary hippocampal neurons were prepared from C57BL/6J ${\mathrm{PV}}^{\mathrm{Cre}}$ knock-in mice express Cre recombinase in PV-expressing neurons as described.^38^ For targeted PV expression of tdTomato, cultures were infected at DIV7 with AAV-FLEX-tdTomato. For WFA staining, cells were incubated for 10 to 15 min in feeding media containing 1:4000 WFA. The experiments were performed in 16 to 21 DIV cultures, at room temperature in a recording chamber on the stage of FV300 inverted confocal microscope (Olympus, Japan). Extracellular Tyrode solution contained (in mM): NaCl, 145; KCl, 3; glucose, 15; HEPES, 10; ${\mathrm{MgCl}}_{2}$, 1.2; ${\mathrm{CaCl}}_{2}$, 1.2; and pH adjusted to 7.4 with NaOH. Synaptic blockers (in mM: 25 DNQX, 50 AP-5, and 10 gabazine) were added to the Tyrode solution. Whole-cell patch clamp internal solution for intrinsic excitability measurements contained (in mM): K-gluconate 135; NaCl 10; ${\mathrm{MgCl}}_{2}$ 2; EGTA 0.5; HEPES 10; Mg-ATP 2; and Na-GTP 0.3.

For intrinsic excitability, frequency was measured by calculating the rate of action potentials in current clamp during 500-ms long depolarizing steps of increasing intensity; a small DC current was injected to maintain membrane potential at −65  mV in between depolarizations. Input resistance ($R_{\text{in}}$) was measured by calculating the slope of the voltage change in response to increasing current injections. Neurons were excluded from the analysis if serial resistance was >20  MΩ. Signals were recorded using MultiClamp 700B amplifier, digitized by DigiData1440A (Molecular Devices, Sunnyvale, California, United States) at 10 kHz, and filtered at 2 kHz. Electrophysiological data were analyzed using pClamp (Molecular Devices).

### 4.9 Statistical Analysis

Prism (version 8.3.0) was used for statistical analysis. The Kolmogorov–Smirnov normality test was used to determine normal distribution of the data and the F-test for determining homogeneity of variance. For normally distributed data with equal variance [Fig. 1(c) and Fig. S4C in the Supplementary Material], we used one-way analysis of variance. For *post* *hoc* analysis, multiple comparisons were corrected using Šídák’s or Tukey’s multiple comparisons test, according to the primary analysis and the software’s recommendation. For normally distributed data with unequal variance [Figs. 1(i) and 3(e)], we used unpaired t-test with Welch’s correction to compare experimental groups. For nonnormally distributed data (Fig. S4D in the Supplementary Material), we used unpaired Mann–Whitney test. To test differences in variance [Fig. 3(e)], we used the Levene’s test. The p-values <5% were considered significant. In all experiments, measurements were taken from distinct samples (different animals) and are presented as median with all individual values.

## Code, Data, and Materials Availability

All code, data, and materials are available in the main text or the supplementary materials. Any additional data will be provided upon request.
