# Supplementary material for: Longitudinal in vivo imaging of perineuronal nets
Source: Neurophotonics. 2023 Mar 24;10(1):015008. doi: 10.1117/1.NPh.10.1.015008 (PMC10037344; doi:10.1117/1.NPh.10.1.015008)
Supplement: Supplementary file 1 [file NPh_010_015008_SD001.pdf]

Supplementary Materials for

Longitudinal *in vivo* imaging of perineuronal nets

Amit Benbenishty, Shany Peled-Hajaj, Venkat Raghavan Krishnaswamy, Hagai Har-Gil, Sapir Havusha-Laufer, Antonella Ruggiero, Inna Slutsky, Pablo Blinder* and Irit Sagi*

*Corresponding authors. Email: pb@tauex.tau.ac.il and irit.sagi@weizmann.ac.il

This PDF file includes:

Figs. S1 to S7 and captions for Videos 1-S4

Other Supplementary Materials for this manuscript include the following:
Videos S1 to S5

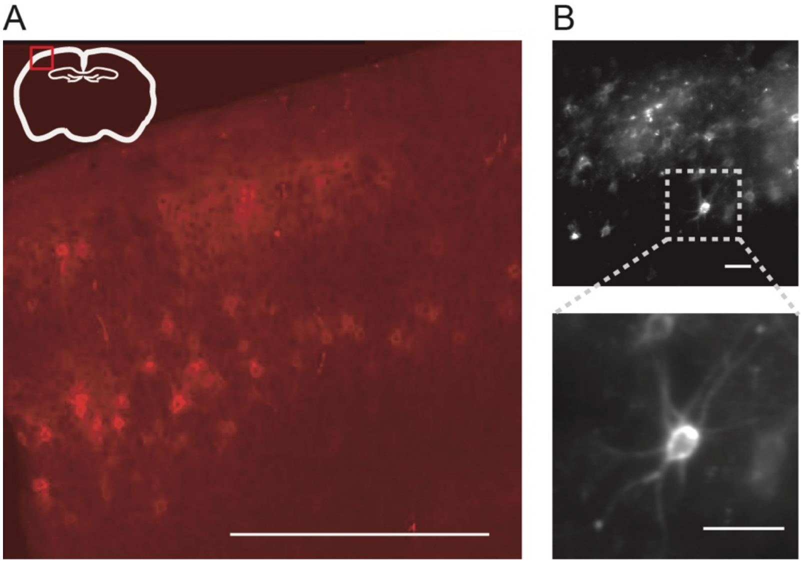

Fig. S1 Assessment of conjugation efficacy. (**A**) Histological staining of PNNs *ex-vivo* using Alexa594-conjugated WFA proved that stainig with this compound was robust and specific. Scale bar is 500 µm. (**B**) *In vivo* injection of WFA-Alexa594 resulted in specific staining of PNNs. Images represent a 30 µm maximum intensity z-projection. Scale bars are 50 µm.

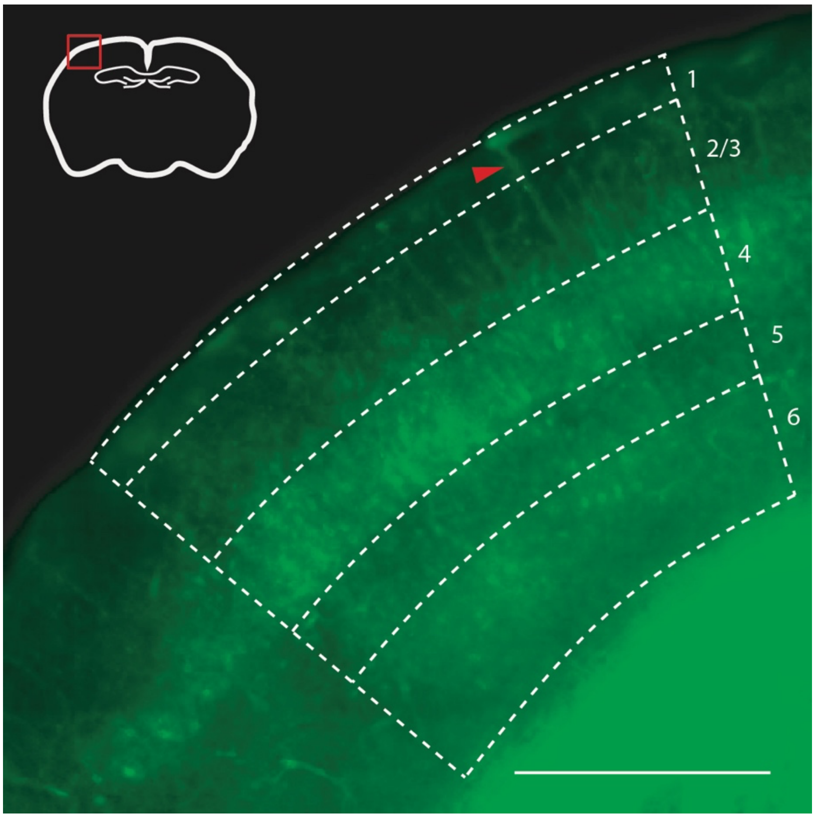

Fig. S2 ***Ex-vivo*** **PNN quantification.** Histological staining of WFA *ex-vivo* using FITC-conjugated WFA. PNNs were counted in the different layers to compare to *in vivo* staning in Fig. 1. Red arrowhead points to a large blood vessel stained by WFA-FITC in layer 1. Scale bar is 500 µm.

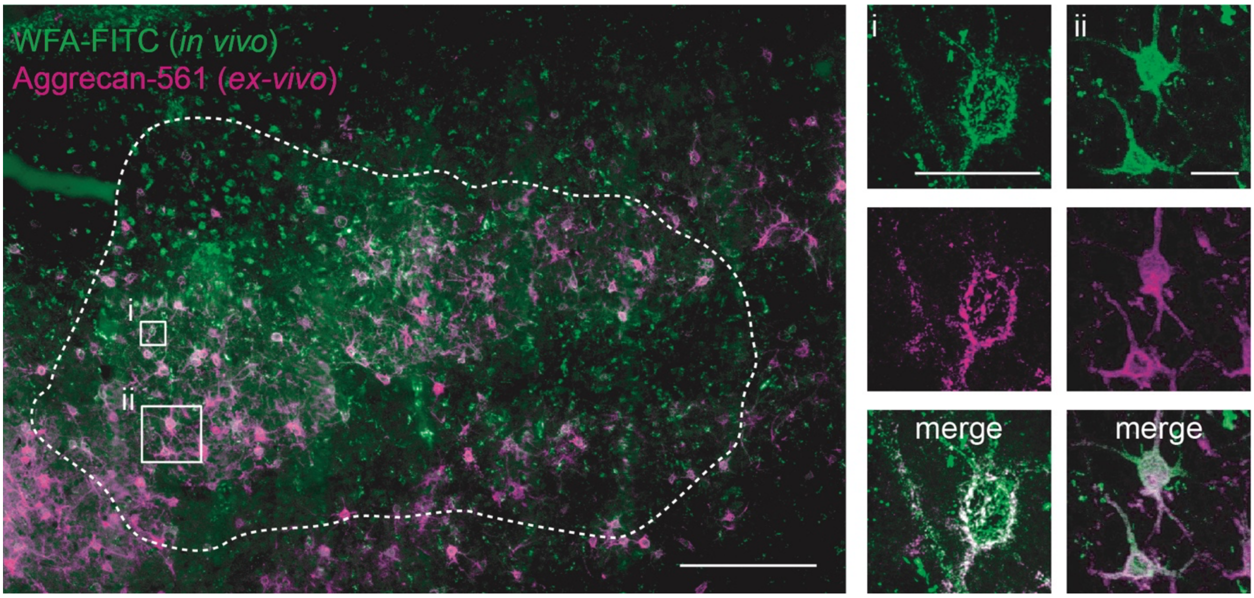

Fig. S3 Asssment of the specificity and efficacy of *in vivo* PNN-labeling. Histological analysis of brains 60 days following WFA-FITC (green) injection revealed that all PNNs around the injection site were labeled, as indicated by *ex-vivo* staining (magenta) with aggrecan. Scale bars are 200 µm and 20 µm for macro image and insets, respectively.

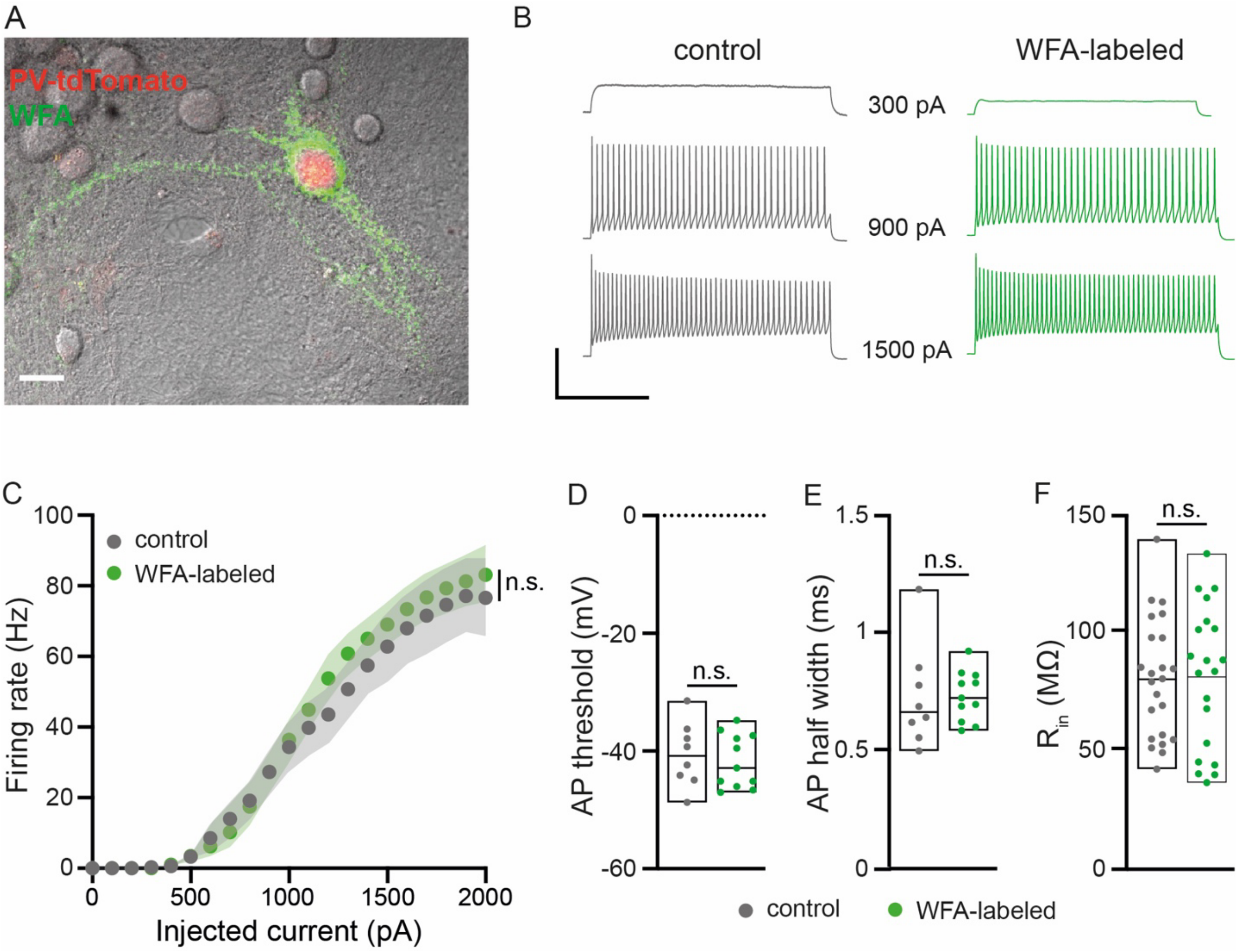

Fig. S4 WFA labeling does not affect intrinsic excitability and membrane resistance of PV-positive fast-spiking interneurons. (**A**) Representative image of a PV^+ neuron expressing tdTomato (red, due to infection of PV-Cre neuronal cultures with AAV-*flex*-tdTomato) labeled with WFA (green). Scale bar is 100 µm. (**B**) Representative traces of voltage responses evoked by current injections in PV-tdTomato expressing neurons (grey) versus neurons stained with WFA (green). Scale bars are 50 mV and 200 ms. (**C**) F-I relationship. No difference (two-way ANOVA, $F_{(1,36)} = 0.2151$, $p = 0.6456$) in firing rate was observed between PV-tdTomato (grey, n = 23) and the ones stained with WFA-stained (green, n = 20) neurons across the entire range of injected currents. (**D**) No difference (unpaired Mann-Whitney test, U = 36, $p = 0.5305$) was found in membrane input resistance between PV-tdTomato (n =8) and WFA-stained (n = 11) neurons. (**E**) No difference (unpaired Mann-Whitney test, U = 35, $p = 0.4920$) was found in AP threshold voltage between PV-tdTomato (n =8) and WFA-stained (n = 11) neurons. (**F**) No difference (unpaired Mann-Whitney test, U = 221, $p = 0.8377$) was found in membrane input resistance between PV-tdTomato (n = 23) and WFA-stained (n = 20) neurons. ns, non-significant. Box plots represent mean with min-max values.

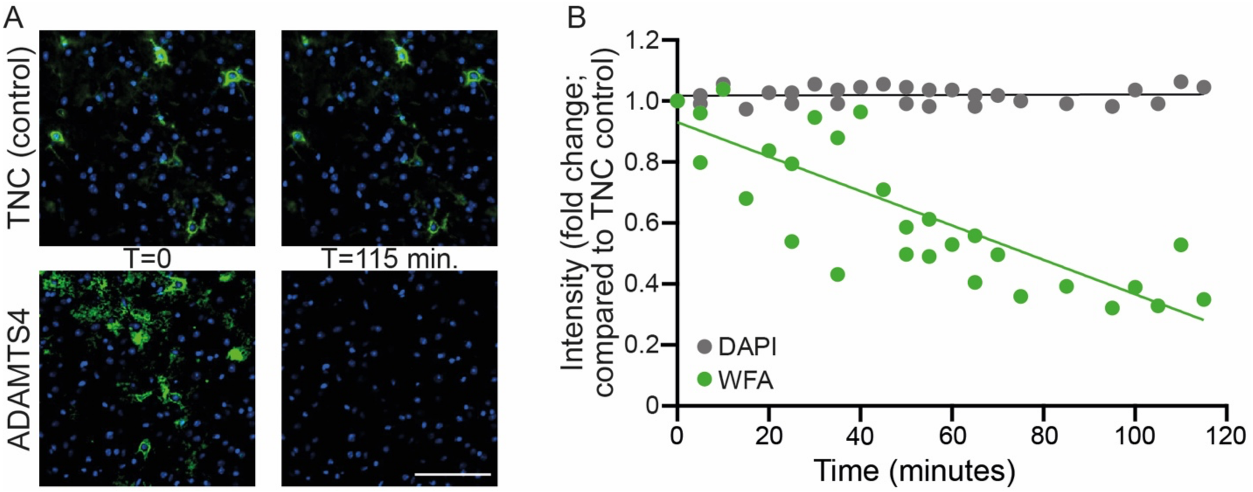

Fig. S5 WFA labeling does not affect enzymatic degradation of PNNs by ADAMTS4 *in vitro*.

(**A**) Representative images of mouse brain slices stained with WFA-FITC (green) and DAPI (blue), treated with ADAMTS4 or TNC (control). Scale bar is 50 µm. (**B**) ADAMTS4 degraded WFA by approximately 70% within two hours (simple linear regression, $F_{(1,20)} = 58.21$, $p < 0.0001$).

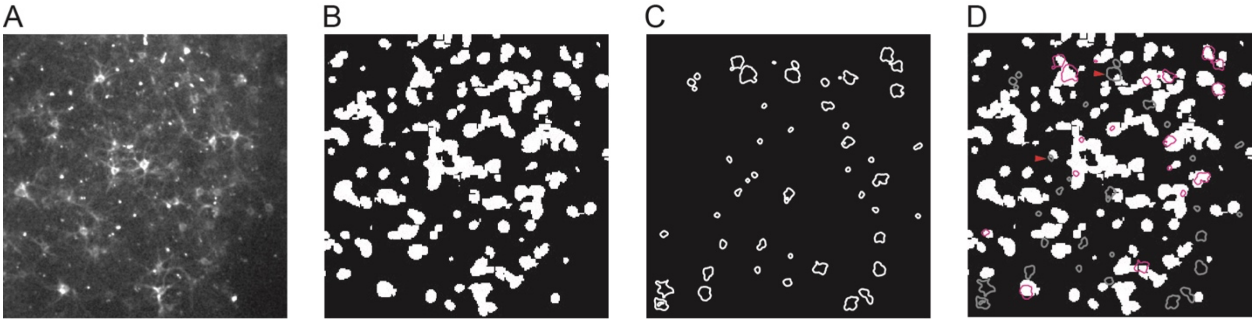

Fig. S6 Representative images for analysis of activity of PV-interneurons with and without PNNs. (**A**) Raw images of the PNN channel were first (**B**) masked (detaild in the methods section). (**C**) ROIs of the traces were then projected on the on to the masked image as seen in (**D**). ROIs with more than 60% covarage were considered PNN$^+$ (magenta), ROI with less than 10% covarage were considered PNN$^-$ (grey), and those with 10-60% were removed from analysis (red arrows).

```
run("Deinterleave", "how=2");
run("Set Scale...", "distance=0 known=0 unit=pixel global");
setOption("ScaleConversions", true);
run("8-bit");
run("Subtract Background...", "rolling=50");
run("Auto Local Threshold", "method=Phansalkar radius=15 parameter_1=0 parameter_2=0 white");
run("Remove Outliers...", "radius=1 threshold=50 which=Bright");
run("Close-");
run("Fill Holes");
setOption("BlackBackground", true);
run("Dilate");
setOption("BlackBackground", true);
run("Dilate");
```

Fig. S7 Fiji macro for masking the PNN channel. The detailed macro allowed masking PNNs only, while substracting the background and noise. To account for movements during awake imaging, the masks were dialated by two pixels.

Videos captions

Video 1
Intravital two-photon imaging of PNNs following intracranial injection of WFA-FITC. A 150 μm stack at depths of 200-350 μm from pial surface (MPEG-4, 1.5MB).

Video 2
Intravital parvalbumin and PNN imaging reconstruction. Intravital two-photon imaging of PNNs (green) in PV^+-tdTomato (magenta) mice following intracranial injection of WFA-FITC. A 100 μm reconstruction at depths of 250-350 μm from pial surface (MOV, 12MB).

Video 3
Intravital PNN-positive and PNN-negative parvalbumin cells. Intravital imaging of cells (magenta) in PV^+-tdTomato mice following intracranial injection of WFA-FITC. One of the PV^+ cells, including part of its dendrites is covered by a PNN (green). A 30 μm stack at depths of 200-230 μm from pial surface (MOV, 11.5MB).

Video 4
Histological reconstruction following *in vivo* fluorescent-WFA injection. *Ex-vivo* imaging of PNNs 60 days following *in vivo* injection of WFA-FITC. A 50 μm reconstruction (MOV, 11.8MB).

Video 5
Calcium imaging following *in vivo* fluorescent-WFA injection. *In vivo* calcium imaging (GCaMP7f; grey) of PV^+ cells with (WFA-594; magenta) and without PNNs. For presentation purposes every 30 frames were averaged and video is presented in 30 frames per second. Scale bar is 20 μm (MOV, 1.6MB).
